# Development of a GRPR-Targeted ^99m^Tc/^188^Re Theranostic Pair Based on NOTA-P2-RM26

**DOI:** 10.3390/pharmaceutics18101247

**Published:** 2026-10-01

**Authors:** Antonio Shegani, Nektarios Pirmettis, Sotiria Triantopoulou, Areti Titoni, Adrianna Travlou, Georgia I. Terzoudi, Minas Papadopoulos, Ioannis Pirmettis, Charalampos Triantis

**Affiliations:** 1 Institute of Nuclear & Radiological Sciences & Technology, Energy & Safety, National Centre for Scientific Research “Demokritos”, 15310 Athens, Greeceiro@rrp.demokritos.gr (S.T.); gterzoudi@rrp.demokritos.gr (G.I.T.); mspap@rrp.demokritos.gr (M.P.);; 2 Department of Pharmacy, Frederick University, 1036 Nicosia, Cyprus; andrianatravlou@gmail.com (A.T.); hsc.tc@frederick.ac.cy (C.T.)

**Keywords:** gastrin-releasing peptide receptor, NOTA-P2-RM26, RM26 antagonist, technetium-99m, rhenium-188, tricarbonyl radiochemistry, SPECT/CT imaging, PC-3 xenografts, prostate cancer, theranostic

## Abstract

**Background:** Gastrin-releasing peptide receptor (GRPR)-targeted radioligands are promising tools for imaging and treatment of GRPR-expressing malignancies. This study aimed to develop a NOTA-P2-RM26-based ^99m^Tc/^188^Re theranostic pair using the *fac*-[M(CO)_3_]^+^ core, with *fac*-[^99m^Tc][Tc(CO)_3_(NOTA-P2-RM26)], ^99m^Tc2, as the diagnostic SPECT analogue and *fac*-[^188^Re][Re(CO)_3_(NOTA-P2-RM26)], ^188^Re2, as the potential therapeutic counterpart. **Methods:** NOTA-P2-RM26 was radiolabeled with the *fac*-[^99m^Tc(CO)_3_(H_2_O)_3_]^+^, ^99m^Tc1, precursor after systematic optimization of peptide amount, pH, buffer, temperature, and reaction time. The radioconjugate was purified by HPLC, reformulated by solid-phase extraction, and evaluated by radio-HPLC and radio-TLC. We assessed stability in the formulation, human serum, cysteine and histidine over 6 h. Lipophilicity was determined as logD_7.4_. We evaluated in vitro specificity, cell association, and internalization in PC-3 cells. We performed biodistribution, blocking, dynamic imaging, and SPECT/CT imaging in PC-3 xenograft-bearing mice. The corresponding ^188^Re analog was prepared under analogous conditions and evaluated for physicochemical matching. **Results:** Optimized labeling afforded ^99m^Tc2 with approximately 85% radiochemical conversion and >97% radiochemical purity after purification. The compound remained stable in formulation, with 93% of the tracer intact after 6 h, and exhibited a hydrophilic profile (logD_7.4_ = −1.5 ± 0.2). In PC-3 cells, uptake reached 20.0 ± 1.0% of added activity and was reduced to 1.4 ± 0.3% under blocking conditions. In vivo, tumor uptake was 15.0 ± 1.0 %IA/g at 30 min and 17.0 ± 2.2 %IA/g at 1 h p.i. Blocking reduced tumor uptake to 0.84 ± 0.12 %IA/g, corresponding to approximately 95% inhibition. Pancreatic uptake decreased from 8.0 ± 0.4 %IA/g at 30 min to 3.0 ± 0.35 %IA/g at 1 h and was reduced to 0.20 ± 0.08 %IA/g after blocking. At 1 h p.i., tumor-to-blood, tumor-to-muscle, tumor-to-pancreas, and tumor-to-kidney ratios were approximately 21.3, 130.8, 5.7, and 3.6, respectively. SPECT/CT imaging confirmed clear, blockable visualization of the PC-3 tumor. The ^188^Re analog was obtained with approximately 80% radiochemical conversion and >95% radiochemical purity post purification. It showed similar stability values to ^99m^Tc2 under the tested conditions and a logD_7.4_ of −1.6 ± 0.2. **Conclusions:** Complex ^99m^Tc2 demonstrated efficient preparation, high stability, GRPR-mediated tumor uptake, favorable tumor-to-background ratios, and clear SPECT/CT visualization of PC-3 xenografts. The corresponding ^188^Re analog supports the feasibility of a chemically matched ^99m^Tc/^188^Re NOTA-P2-RM26 theranostic platform and warrants further evaluation of ^188^Re biodistribution, dosimetry, and therapeutic efficacy.

## 1. Introduction

Gastrin-releasing peptide receptor (GRPR), also known as bombesin receptor subtype 2, is a G-protein-coupled receptor that has attracted considerable interest as a molecular target for cancer imaging and radionuclide therapy [1]. GRPR is physiologically expressed in selected normal tissues, including the gastrointestinal tract and pancreas, but it is also overexpressed in several malignancies, particularly prostate and breast cancer [2,3,4]. In prostate cancer, GRPR-targeted imaging has been investigated as a complementary approach to established molecular imaging strategies, with the potential to visualize GRPR-positive lesions and assess receptor expression in vivo [5,6,7].

Radiolabeled bombesin analogs were initially developed mainly as GRPR agonists. However, antagonist-based radioligands have gained increasing attention because they can provide high tumor uptake while avoiding receptor activation-related physiological effects. Preclinical evidence supports a shift toward GRPR antagonists, showing that bombesin receptor antagonists may offer superior tumor-targeting properties compared with agonists [8]. Among the antagonist scaffolds developed for GRPR targeting, RM26, with the sequence D-Phe-Gln-Trp-Ala-Val-Gly-His-Sta-Leu-NH_2_, has shown high affinity for GRPR and has been widely explored as a platform for PET, SPECT, and therapeutic radioconjugates [7,9,10,11,12].

NOTA-P2-RM26 or NOTA-PEG_2_-RM26 contains the RM26 antagonist sequence connected to NOTA through a diethylene glycol spacer. This scaffold has previously been evaluated with PET radionuclides, while ^188^Re-labeled RM26 derivatives have been explored using alternative chelation strategies [13]. The [^18^F]AlF-labeled analog demonstrated specific binding to PC-3 cells, low internalization, rapid blood clearance, low liver uptake, and favorable tumor-to-background ratios in PC-3 xenograft-bearing mice [10]. In addition, researchers have investigated ^68^Ga-labeled NOTA-P2-RM26 and related RM26 analogs in preclinical and clinical settings, supporting the translational relevance of this ligand family for GRPR-targeted imaging [11,12]. More recently, first-in-human experience with [^68^Ga]Ga-NOTA-PEG_2_-RM26 further confirmed the clinical interest in this scaffold for imaging GRPR-expressing prostate and breast cancer [7].

Although PET-based GRPR imaging has been extensively investigated, ^99m^Tc remains highly attractive for routine nuclear medicine practice due to its broad availability, favorable nuclear properties, and compatibility with SPECT. In parallel, ^188^Re is an appealing therapeutic counterpart because it can be obtained from a ^188^W/^188^Re generator [14,15,16,17] and emits high-energy β^−^ particles suitable for radionuclide therapy, together with a γ emission that may support imaging and dosimetry [18,19]. Recent work with maSSS/maSES-PEG_2_-RM26 demonstrated the feasibility of developing ^99m^Tc/^188^Re RM26-based theranostic pairs and specifically addressed whether ^99m^Tc analogs can be used to estimate the biodistribution and dosimetry of their ^188^Re counterparts [20]. However, these reported RM26 systems relied on N_3_S-type chelators, whereas the present work investigates a distinct *fac*-[M(CO)_3_]^+^-NOTA-P2-RM26 approach.

Several ^99m^Tc-labeled GRPR antagonists have already been reported, including [^99m^Tc]Tc-DB15 and RM26-based maSSS/maSES derivatives, which showed GRPR-specific uptake, tumor visualization by SPECT/CT, and translational potential for clinical imaging [20,21,22]. Early clinical feasibility of ^99m^Tc-labeled GRPR imaging was demonstrated with [^99m^Tc]Tc-RP527, although pharmacokinetic limitations stimulated further development of improved SPECT tracers [23]. Previous ^188^Re-RM26 studies, including MAG_3_-RM26 and maSSS/maSES-PEG_2_-RM26 derivatives, have demonstrated the feasibility of rhenium-based RM26 radioconjugates, although these systems used chelation strategies different from the *fac*-[M(CO)_3_]^+^-NOTA approach investigated here [13,20]. This concept follows the broader development of GRPR antagonist-based theranostic systems, including ^68^Ga/^177^Lu-NeoBOMB1 and ^68^Ga/^177^Lu-RM2-type platforms [24,25].

The *fac*-[M(CO)_3_]^+^ core, where M is technetium or rhenium, provides a well-established organometallic platform for the development of radiolabeled biomolecules [26,27,28]. The precursor *fac*-[^99m^Tc][Tc(CO)_3_(H_2_O)_3_]^+^, ^99m^Tc1, can be prepared directly from generator-eluted [^99m^Tc]TcO_4_^−^ and has been widely used for biomolecule labeling, while analogous rhenium tricarbonyl chemistry enables the preparation of matched Re-containing counterparts [29,30]. Hydrophilic tricarbonyl complexes based on NOTA and NODAGA have previously been reported for ^99m^Tc and ^186^Re, including GRPR-targeted bioconjugates, demonstrating that this chelation strategy can provide stable radioconjugates with favorable in vitro and in vivo properties [31,32]. In particular, Makris et al. reported ^99m^Tc- and ^186^Re-labeled NOTA/NODAGA GRPR antagonist bioconjugates and demonstrated matched-pair targeting of GRPR-expressing PC-3 tumors, establishing an important precedent for the application of NOTA/NODAGA-based tricarbonyl Tc/Re chemistry in GRPR-targeted radiopharmaceutical development [32]. Previous work involving Shegani and co-workers has also contributed to the development of GRPR-targeted radiometal bioconjugates and Re/Tc tricarbonyl radiochemistry platforms [33,34].

Despite these advances, to the best of our knowledge, the direct labeling of NOTA-P2-RM26 with the *fac*-[^99m^Tc(CO)_3_]^+^ core has not yet been reported. This approach combines a clinically relevant RM26 antagonist scaffold with ^99m^Tc tricarbonyl chemistry, enabling the preparation of a chemically matched ^99m^Tc/^188^Re theranostic pair. In the present study, we describe the development of *fac*-[^99m^Tc][Tc(CO)_3_(NOTA-P2-RM26)], ^99m^Tc2, including radiolabeling optimization, HPLC purification, SPE-based reformulation, quality control, stability, lipophilicity, in vitro GRPR specificity, cell-association/internalization profile, biodistribution, blocking studies and SPECT/CT imaging in PC-3 xenograft-bearing mice. In parallel, we prepared and evaluated the corresponding *fac*-[^188^Re][Re(CO)_3_(NOTA-P2-RM26)] analog, ^188^Re2, for stability and lipophilicity to assess the feasibility of this system as a GRPR-targeted ^99m^Tc/^188^Re theranostic platform. To the best of our knowledge, this is the first report of a chemically matched *fac*-[^99m^Tc/^188^Re(CO)_3_]^+^ pair based on NOTA-P2-RM26 for GRPR-targeted radiopharmaceutical development.

## 2. Results

### 2.1. Synthesis and Characterization of ^nat^Re2

**Figure 1:**
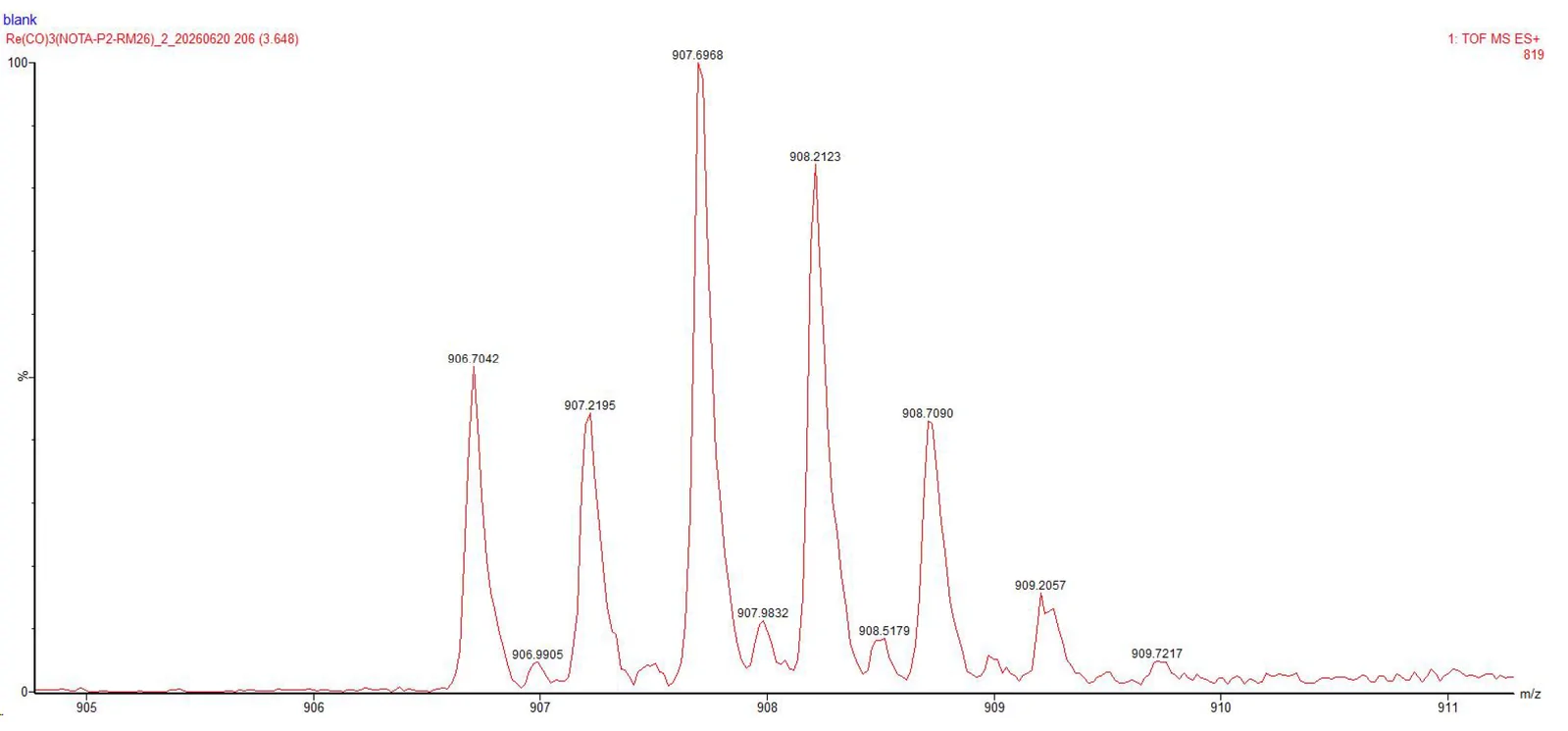
UPLC-QToF-MS characterization of ^nat^Re2. ESI^+^ mass spectrum showing the doubly charged isotope envelope assigned to [M+2H]^2+^ of ^nat^Re2, with peaks at *m*/*z* 906.7042, 907.2195, 907.6968, 908.2123, 908.7090, 909.2057, and 909.7217. The approximately 0.5 *m*/*z* isotope spacing is consistent with a 2+ charge state.

**Scheme 1:**
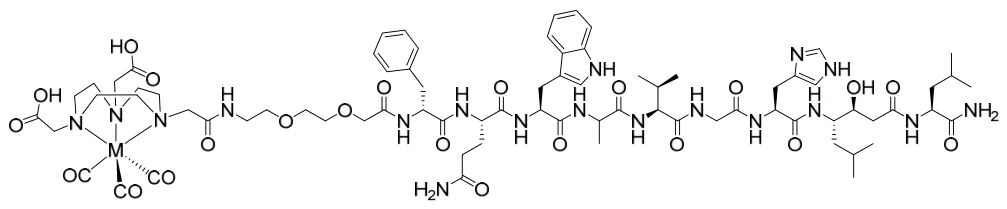
Schematic representation of *fac*-[M(CO)_3_(NOTA-P2-RM26)], M = ^99m^Tc, ^nat^Re or ^188^Re.

The non-radioactive rhenium analog, ^nat^Re2, was synthesized from the corresponding rhenium tricarbonyl precursor, ^nat^Re1, and served as a structural reference for the radiolabeled analogs. The reaction afforded the desired metalated peptide as the main product (Scheme 1), as confirmed by analytical HPLC. The identity of ^nat^Re2 was supported by UPLC-QToF-MS analysis in ESI positive mode. The spectrum showed a doubly charged isotope envelope at *m*/*z* 906.7042, 907.2195, 907.6968, 908.2123, 908.7090, 909.2057 and 909.7217, with approximately 0.5 *m*/*z* isotope spacing, consistent with the expected 2+ charge state and rhenium-containing isotope distribution of C_76_H_110_N_18_O_22_Re (Figure 1). Because the spectrum did not support a sufficiently accurate singly charged exact-mass assignment, it is reported as supportive isotope-pattern evidence rather than as definitive high-resolution exact-mass confirmation. Analytical HPLC showed a retention time of 7.7 min for ^nat^Re2. Comparative HPLC analysis of ^nat^Re2 with the corresponding radiolabeled analogs showed comparable retention behavior, supporting the formation of ^99m^Tc2 and ^188^Re2.

### 2.2. Radiolabeling Optimization of ^99m^Tc2

The *fac*-[^99m^Tc][Tc(CO)_3_(H_2_O)_3_]^+^, ^99m^Tc1, precursor was prepared according to established procedures and used for the radiolabeling of NOTA-P2-RM26. We systematically optimized conditions to efficiently form ^99m^Tc2. The evaluated parameters included peptide amount, buffer composition, pH, reaction temperature, and reaction time (Table 1). We tested peptide amounts of 1, 10, and 100 μg. We performed reactions at room temperature, 75 °C, or 100 °C, with incubation times of 15, 30, and 60 min. We evaluated the effect of pH using 0.1 M sodium acetate buffer at pH 5, 0.1 M phosphate buffer at pH 7 or 9, and non-buffered conditions. All reactions were performed under nitrogen. The optimized conditions were identified as incubation of 100 μg of NOTA-P2-RM26 with freshly prepared ^99m^Tc1 in 0.1 M sodium acetate buffer at pH 5 for 30 min at 75 °C. Under these conditions, the radiochemical conversion (RCC) reached approximately 85%. Increasing the reaction time to 60 min resulted in only a minor increase in RCC and was therefore not considered advantageous for routine preparation. Reflux or heating at 100 °C did not improve conversion compared with 75 °C.

### 2.3. Purification, Reformulation, and Quality Control

Following radiolabeling, the crude reaction mixture was purified by radio-HPLC to isolate ^99m^Tc2. The collected radioactive fraction was subsequently processed using an HLB cartridge for solvent exchange and concentration. The purified radioconjugate was reformulated in saline containing 10% ethanol (*v*/*v*), yielding a final preparation suitable for in vitro and in vivo evaluation.

**Figure 2:**
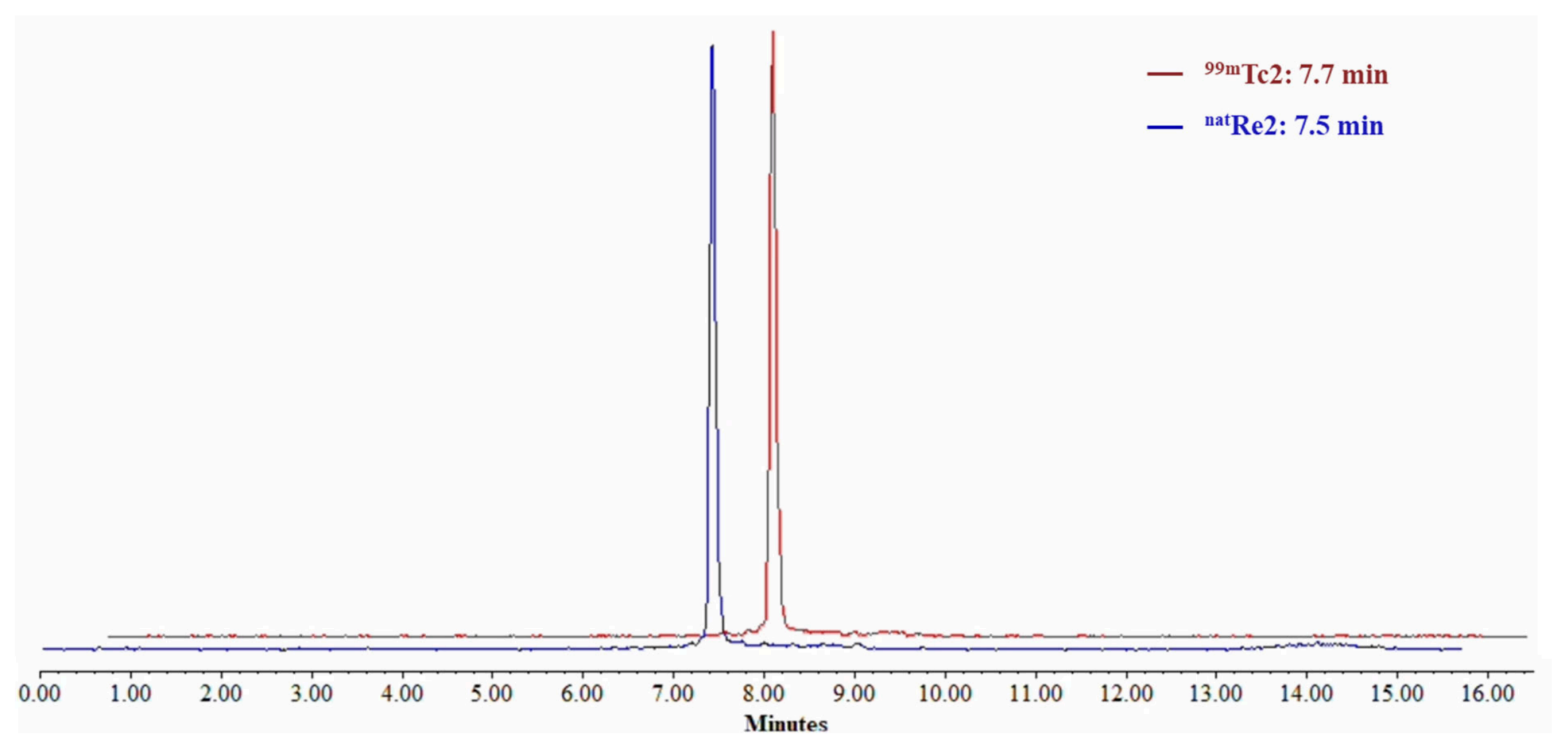
Representative radio-HPLC chromatogram of ^nat^Re2 and ^99m^Tc2.

**Figure 3:**
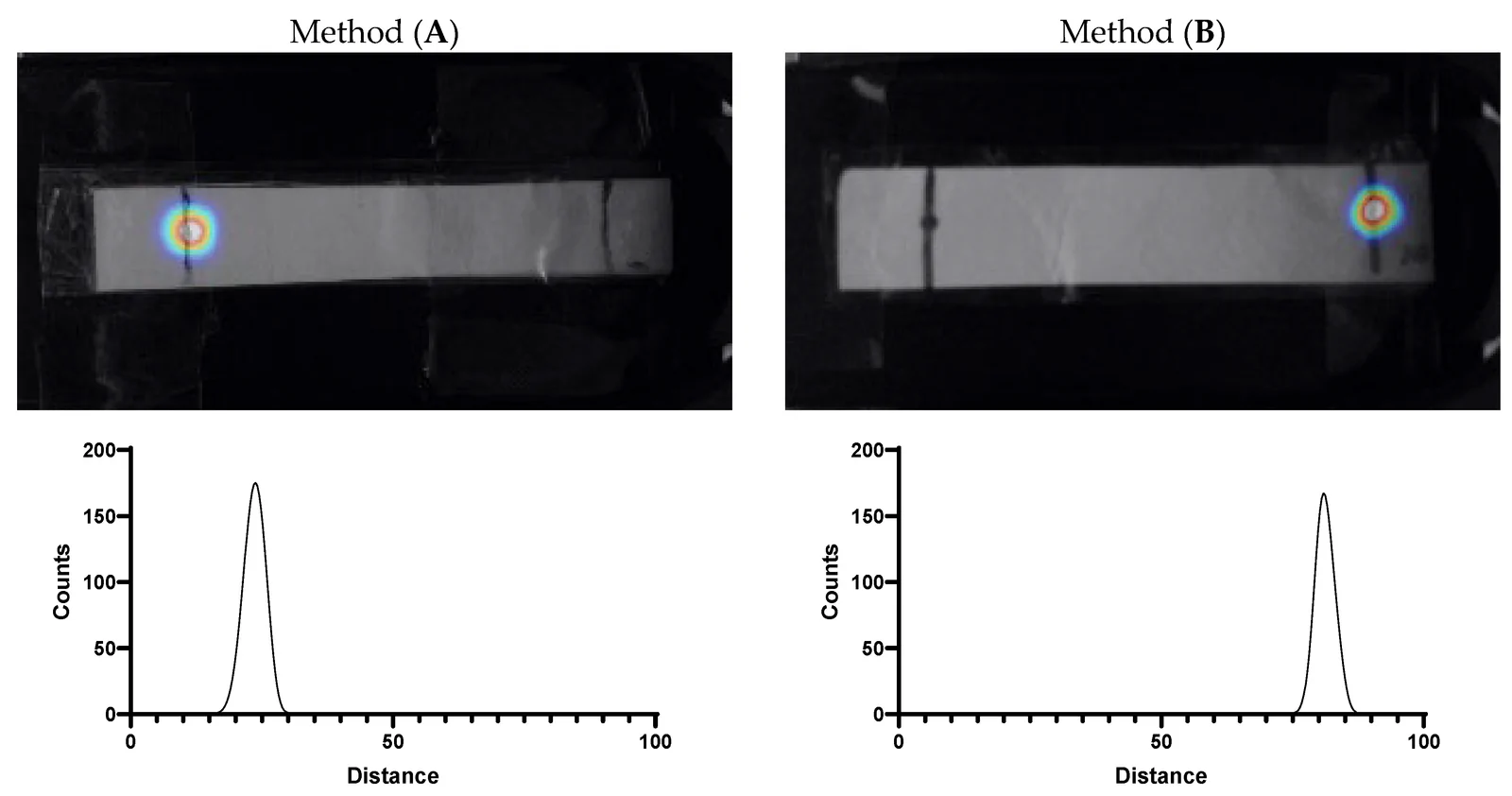
Representative radio-TLC profile of ^99m^Tc2 analyzed using the γ-eye imaging system. Mobile phases: (**A**) saline; (**B**) saline/methanol (1:1, *v*/*v*). (Distance in mm).

Quality control was performed by analytical radio-HPLC and radio-TLC (Figure 2 and Figure 3). Radio-HPLC analysis demonstrated a dominant product peak corresponding to the purified radioconjugate, with radiochemical purity exceeding 97%. Radio-TLC analysis confirmed the absence of significant free pertechnetate or colloidal species. The final radioconjugate was used in biological experiments only when radiochemical purity exceeded 95%.

### 2.4. In Vitro Stability and Lipophilicity of ^99m^Tc2

**Figure 4:**
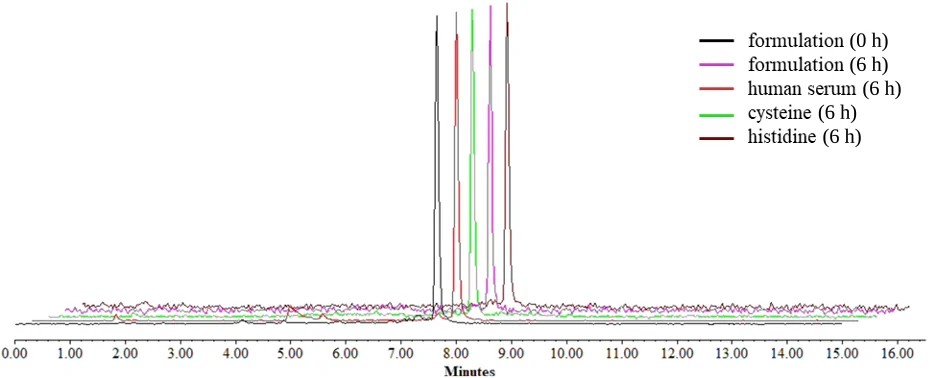
Stability profile of ^99m^Tc2 in formulation, human serum, cysteine, and histidine after incubation for up to 6 h at 37 °C.

The in vitro stability of ^99m^Tc2 was evaluated in the final formulation, in human serum, and in the presence of cysteine and histidine for up to 6 h at 37 °C (Figure 4 and Table 2). In the final formulation containing 10% ethanol (*v*/*v*) in saline, the radioconjugate remained stable, with radiochemical purity decreasing from 99% at 1 h to 93% at 6 h.

In human serum, the radiochemical purity decreased from 92% at 1 h to 85% at 6 h. Challenge studies showed that ^99m^Tc2 remained highly stable in the presence of cysteine, with 92% of the intact radioconjugate remaining after 6 h. In contrast, incubation with histidine moderately decreased radiochemical purity to 85% at 6 h. This decrease was more pronounced in histidine and human serum than in cysteine, suggesting partial susceptibility to challenge under nitrogen-donor or protein-containing conditions.

We determined the distribution coefficient using the shake-flask method in n-octanol/PBS at pH 7.4. ^99m^Tc2 showed a logD_7.4_ value of −1.5 ± 0.2, indicating a hydrophilic profile.

### 2.5. Radiolabeling and Physicochemical Evaluation of ^188^Re2

The corresponding ^188^Re2 analog was prepared using the *fac*-[^188^Re][Re(CO)_3_(H_2_O)_3_]^+^ (^188^Re1) precursor, which was synthesized according to established procedures in the literature [30,35]. We radiolabeled NOTA-P2-RM26 under optimized conditions developed for ^99m^Tc2: 0.1 M sodium acetate buffer at pH 5, 75 °C, and 30 min incubation. Under these conditions, ten independent preparations gave an RCC of approximately 80%. Following HPLC purification and SPE-based reformulation, the RCP exceeded 95% in all preparations. Further optimization did not substantially improve the conversion. Radio-HPLC showed retention behavior comparable to the non-radioactive rhenium reference compound and the ^99m^Tc analog.

The stability of ^188^Re2 was evaluated in formulation, human serum, cysteine and histidine at 1, 3 and 6 h. The RCP values obtained at 1, 3, and 6 h under the different incubation conditions are presented in Table 3. It was also hydrophilic, with logD_7.4_ = −1.6 ± 0.2. These data support the physicochemical compatibility of ^99m^Tc2 and ^188^Re2 as a chemically matched diagnostic/therapeutic pair.

### 2.6. In Vitro GRPR Specificity in PC-3 Cells

**Figure 5:**
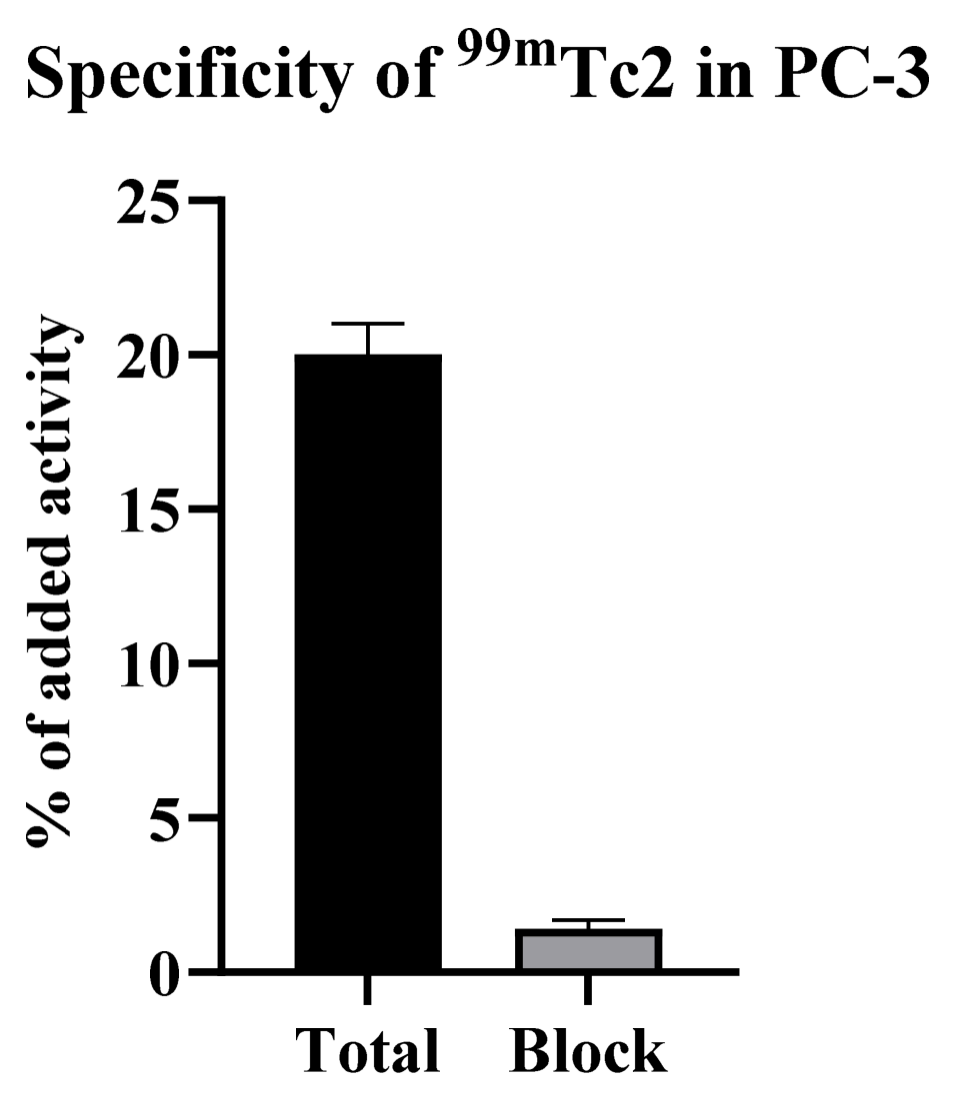
In vitro specificity of ^99m^Tc2 in PC-3 cells. Cell-associated activity was measured after 1 h incubation at 37 °C in the absence or presence of excess NOTA-P2-RM26. Data are presented as mean ± SD from three independent experiments. Statistical comparison was performed using an unpaired two-tailed Student’s *t*-test; *p* < 0.0001.

The receptor specificity of ^99m^Tc2 was evaluated in GRPR-positive PC-3 cells by competitive blocking with an excess of non-radioactive NOTA-P2-RM26 (Figure 5). After 1 h of incubation at 37 °C, ^99m^Tc2 showed high cell-associated activity, reaching 20.0 ± 1.0% of the added activity. Co-incubation with excess NOTA-P2-RM26 markedly reduced cell-associated activity to 1.4 ± 0.3%, corresponding to approximately 93% inhibition of uptake. This reduction was statistically significant (*p* < 0.0001) and confirms that cellular uptake of ^99m^Tc2 in PC-3 cells is predominantly GRPR-mediated.

### 2.7. Cell-Association and Internalization Profile in PC-3 Cells

**Figure 6:**
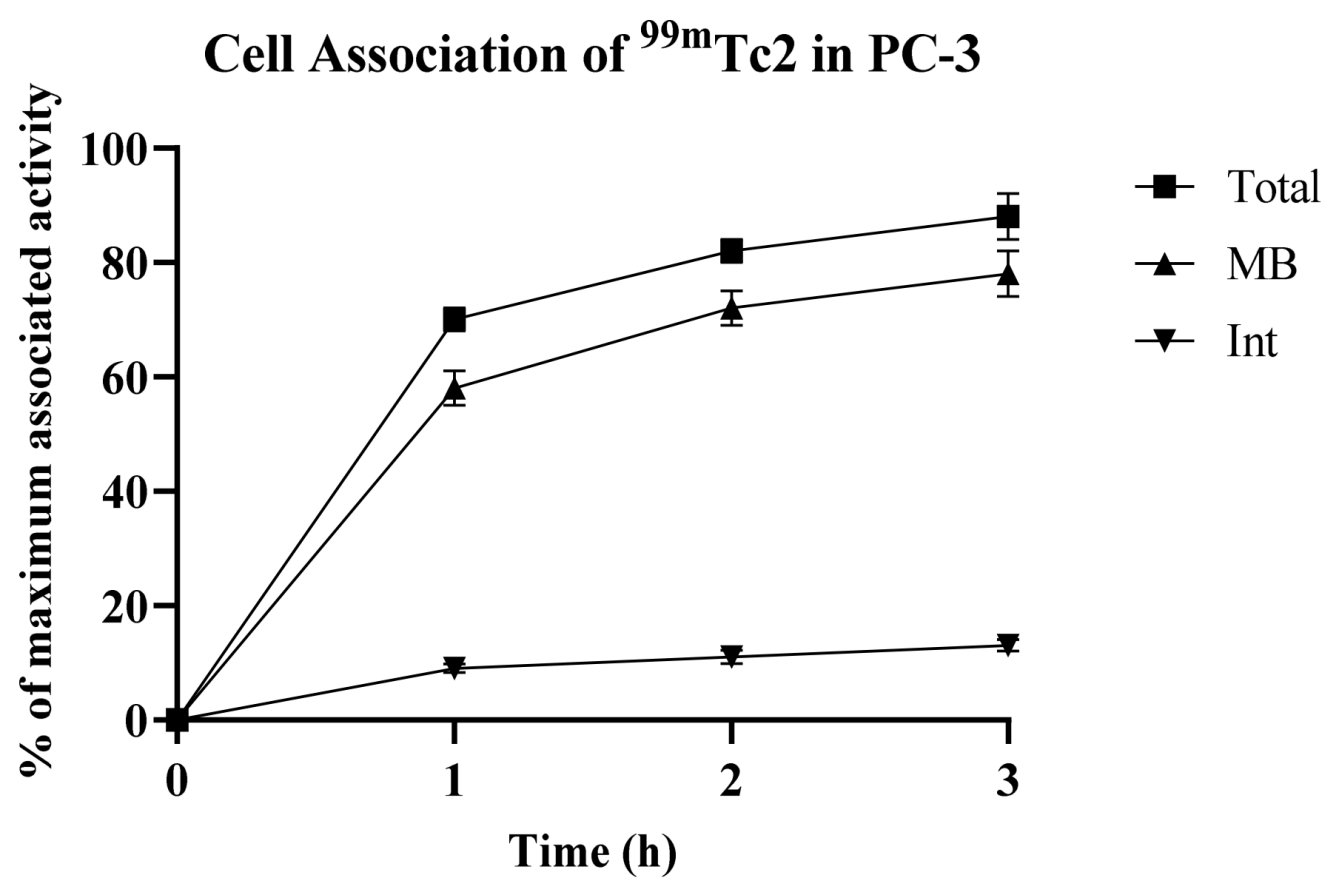
Time-dependent cell-association profile of ^99m^Tc2 in PC-3 cells. Total cell-associated activity, membrane-bound fraction, and internalized fraction are shown over time. MB: membrane-bound fraction; Int: internalized fraction. Data are presented as mean ± SD from three independent experiments.

The time-dependent cell-association profile of ^99m^Tc2 was evaluated in PC-3 cells over 3 h (Figure 6). Total cell-associated activity increased rapidly during the first hour, reaching 70.0 ± 2.0% of the maximum, then continued to increase gradually, reaching 88.0 ± 4.0% at 3 h. Fractionation of the cell-associated radioactivity into membrane-bound and internalized components showed that most of the activity remained associated with the cell membrane throughout the experiment. At 1 h, the membrane-bound fraction accounted for 58.0 ± 3.0% of the maximum associated activity, whereas the internalized fraction represented 12.0 ± 0.8%. At 3 h, membrane-associated activity remained dominant at 78.0 ± 4.0%, while the internalized fraction remained comparatively low at 10.0 ± 1.0%. When expressed relative to total cell-associated activity, the membrane-bound fraction accounted for approximately 83% at 1 h and 89% at 3 h, whereas the internalized fraction accounted for approximately 17% and 11%, respectively. This profile is consistent with the expected behavior of RM26-based GRPR antagonists, which typically show strong receptor binding with limited internalization.

### 2.8. Biodistribution in PC-3 Xenograft-Bearing Mice

**Figure 7:**
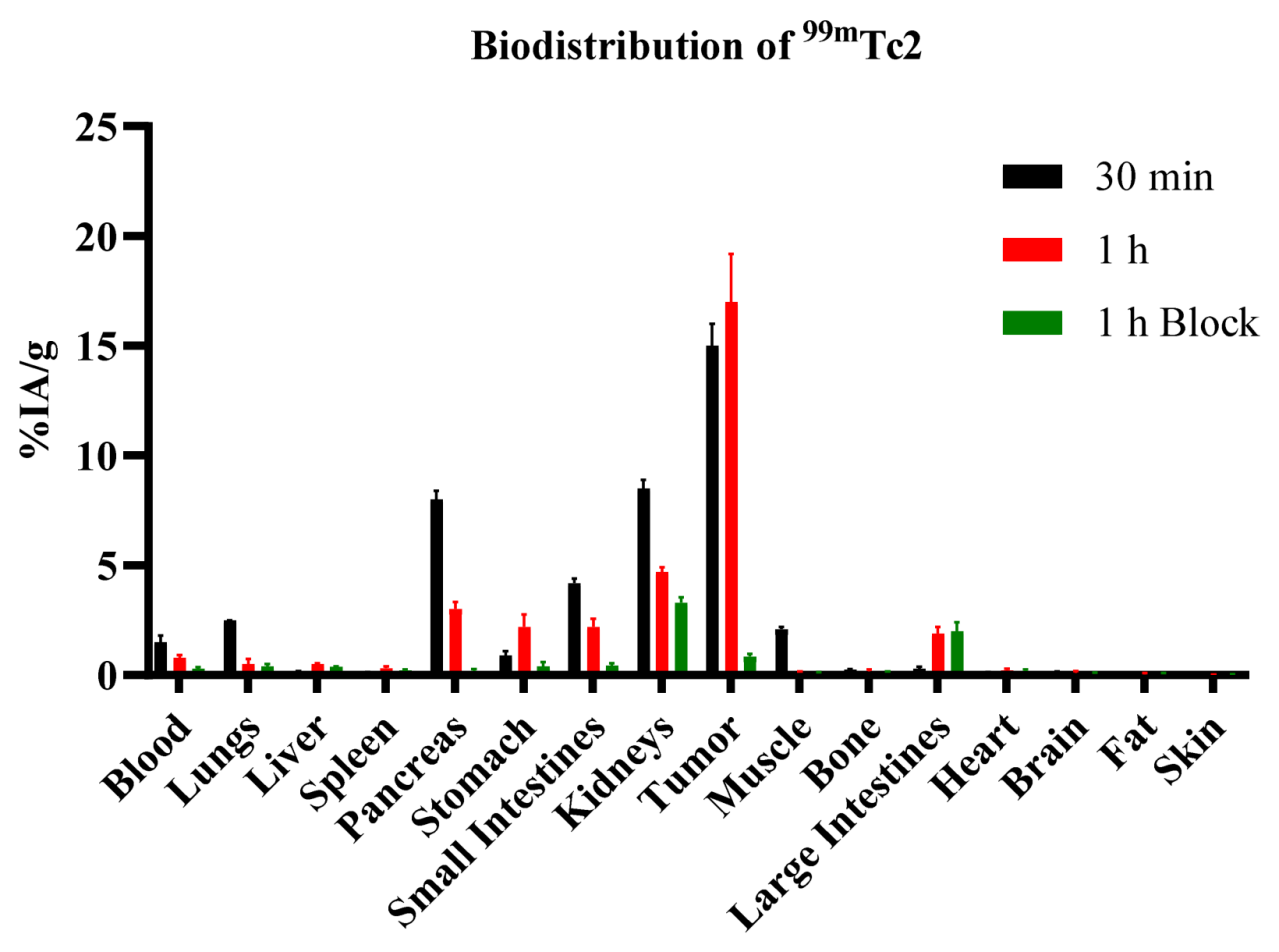
Biodistribution of ^99m^Tc2 in PC-3 xenograft-bearing mice at 30 min, 1 h, and 1 h after blocking with excess NOTA-P2-RM26. Data are expressed as %IA/g. The stomach and intestines are reported to be empty.

We evaluated the biodistribution of ^99m^Tc2 in PC-3 xenograft-bearing mice at 30 min and 1 h post injection (p.i.). A receptor-blocking group was also evaluated at 1 h p.i. by administering an excess of NOTA-P2-RM26. Biodistribution data are expressed as %IA/g, except for urine/bladder and gastrointestinal content, which are expressed as %IA (Figure 7). The radioconjugate cleared rapidly from the blood and several non-target tissues. Blood activity decreased from 1.5 ± 0.3 %IA/g at 30 min p.i. to 0.8 ± 0.13 %IA/g at 1 h p.i. A marked decrease was also observed in the lungs, from 2.5 ± 0.02 %IA/g at 30 min to 0.5 ± 0.24 %IA/g at 1 h. Uptake in most non-target tissues remained low at 1 h p.i., including liver (0.5 ± 0.05 %IA/g), spleen (0.33 ± 0.08 %IA/g), muscle (0.13 ± 0.04 %IA/g), bone (0.18 ± 0.07 %IA/g), heart (0.22 ± 0.08 %IA/g), brain (0.10 ± 0.10 %IA/g), fat (0.03 ± 0.07 %IA/g), and skin (0.03 ± 0.01 %IA/g). Tumor uptake was high and retained over the evaluated time interval, reaching 15.0 ± 1.0 %IA/g at 30 min and 17.0 ± 2.2 %IA/g at 1 h p.i. At 1 h p.i., the corresponding tumor-to-blood, tumor-to-muscle, tumor-to-pancreas, and tumor-to-kidney ratios were approximately 21.3, 130.8, 5.7, and 3.6, respectively. Pancreatic uptake was elevated, consistent with the high physiological GRPR expression of this organ in mice. Pancreatic activity was 8.0 ± 0.4 %IA/g at 30 min and decreased to 3.0 ± 0.35 %IA/g at 1 h p.i. Importantly, tumor uptake exceeded pancreatic uptake by approximately 1.9-fold at 30 min and 5.7-fold at 1 h p.i. Other GRPR-expressing tissues, including the stomach and small intestine, showed lower uptake than the tumor, with values of 2.2 ± 0.55 %IA/g and 2.2 ± 0.38 %IA/g, respectively, at 1 h p.i. Kidney uptake was prominent at early time points, decreasing from 8.5 ± 0.4 %IA/g at 30 min to 4.7 ± 0.22 %IA/g at 1 h p.i. Intestinal uptake was also observed, with small-intestine activity decreasing from 4.2 ± 0.2 %IA/g at 30 min to 2.2 ± 0.38 %IA/g at 1 h, while large-intestine activity increased from 0.3 ± 0.08 %IA/g to 1.9 ± 0.3 %IA/g over the same interval. Urine/bladder content increased from 3.5% IA at 30 min to 7.5% IA at 1 h p.i. In contrast, gastrointestinal content accounted for the largest fraction of recovered activity, increasing from 67% IA at 30 min to 82% IA at 1 h p.i. These findings indicate predominant hepatobiliary/intestinal excretion, with a renal contribution.

### 2.9. In Vivo Blocking Studies

In vivo receptor specificity was evaluated by administration of excess NOTA-P2-RM26. Blocking resulted in marked suppression of tumor uptake at 1 h p.i., reducing tumor-associated activity from 17.0 ± 2.2 %IA/g to 0.84 ± 0.12 %IA/g. This corresponds to approximately 95% inhibition of tumor uptake and confirms that ^99m^Tc2 tumor accumulation is predominantly GRPR-mediated. Blocking also strongly reduced pancreatic uptake from 3.0 ± 0.35 %IA/g to 0.20 ± 0.08 %IA/g, corresponding to approximately 93% inhibition. Uptake in the stomach and small intestines was also reduced under blocking conditions, from 2.2 ± 0.55 %IA/g to 0.4 ± 0.2 %IA/g and from 2.2 ± 0.38 %IA/g to 0.44 ± 0.12 %IA/g, respectively. In contrast, uptake in non-target tissues such as blood, liver, spleen, muscle, bone, heart, brain, fat, and skin remained low under blocking conditions. Kidney uptake decreased from 4.7 ± 0.22 %IA/g at 1 h p.i. to 3.3 ± 0.25 %IA/g in the blocking group, indicating that renal uptake was only partially affected by receptor blockade and is therefore likely related mainly to clearance rather than GRPR-specific binding.

### 2.10. Imaging Studies

**Figure 8:**
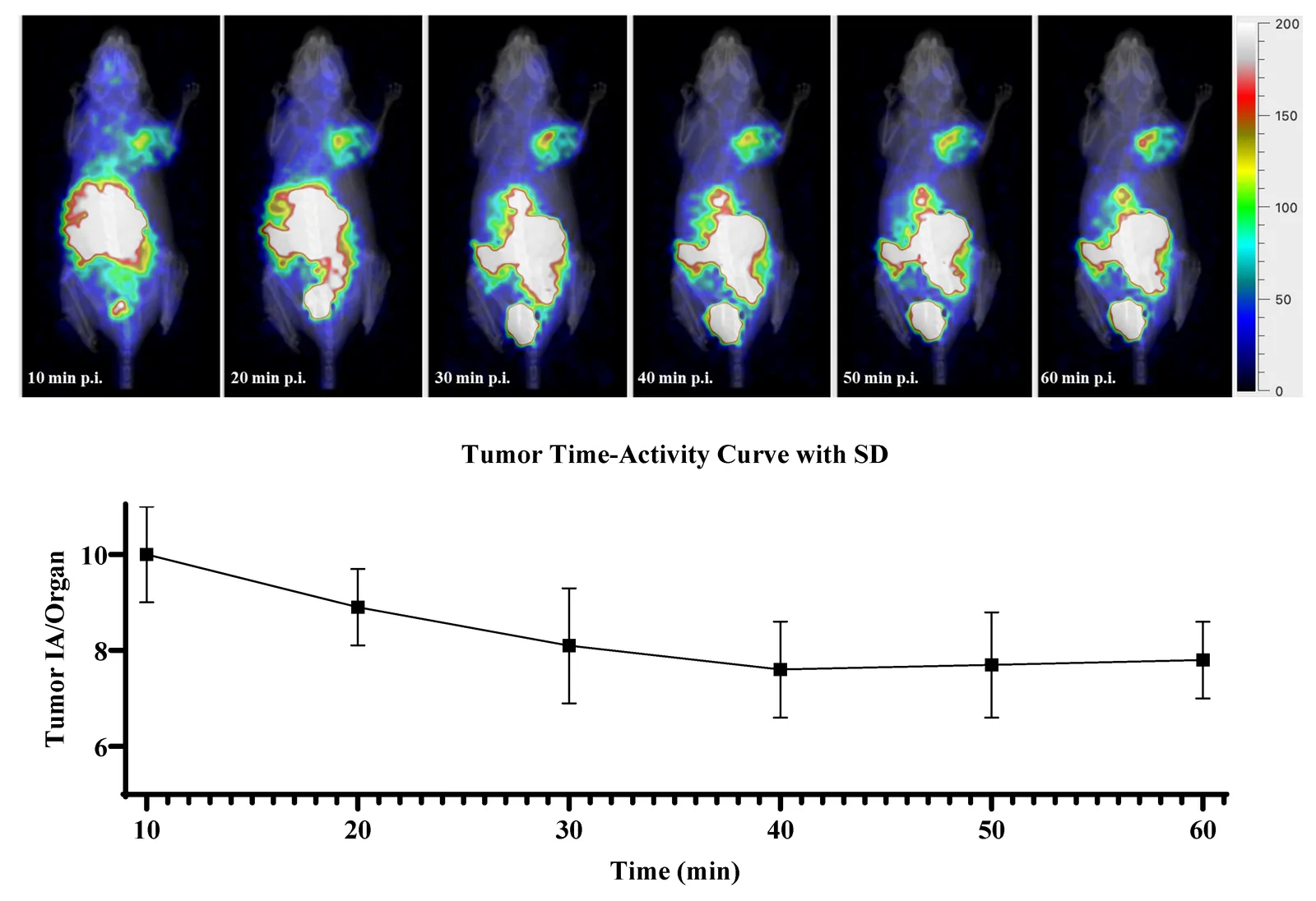
(**Top**): Representative dynamic γ-eye images and time–activity curves of ^99m^Tc2 in PC-3 xenograft-bearing mice up to 1 h p.i. (10, 20, 30, 40, 50 and 60 min). The animals received approximately 30 MBq of radiotracer, and we performed a dynamic 2D acquisition for 60 min. The scale ranges from 0 to 200 counts, with dark blue/black indicating background or low signal and white indicating the highest signal intensity. (**Bottom**): Tumor time-activity curve expressed as %IA/organ. Data are the mean ± SD for three mice.

Dynamic imaging was performed for up to 1 h after injection using the *γ*-eye™ system from Bioemtech (Figure 8). The dynamic acquisition showed early systemic distribution followed by progressive clearance of radioactivity. Prominent abdominal activity was observed during the acquisition, consistent with the hepatobiliary/intestinal excretion pattern identified by ex vivo biodistribution. Tumor-associated activity was detectable during the imaging sequence, while background activity decreased over time.

**Figure 9:**
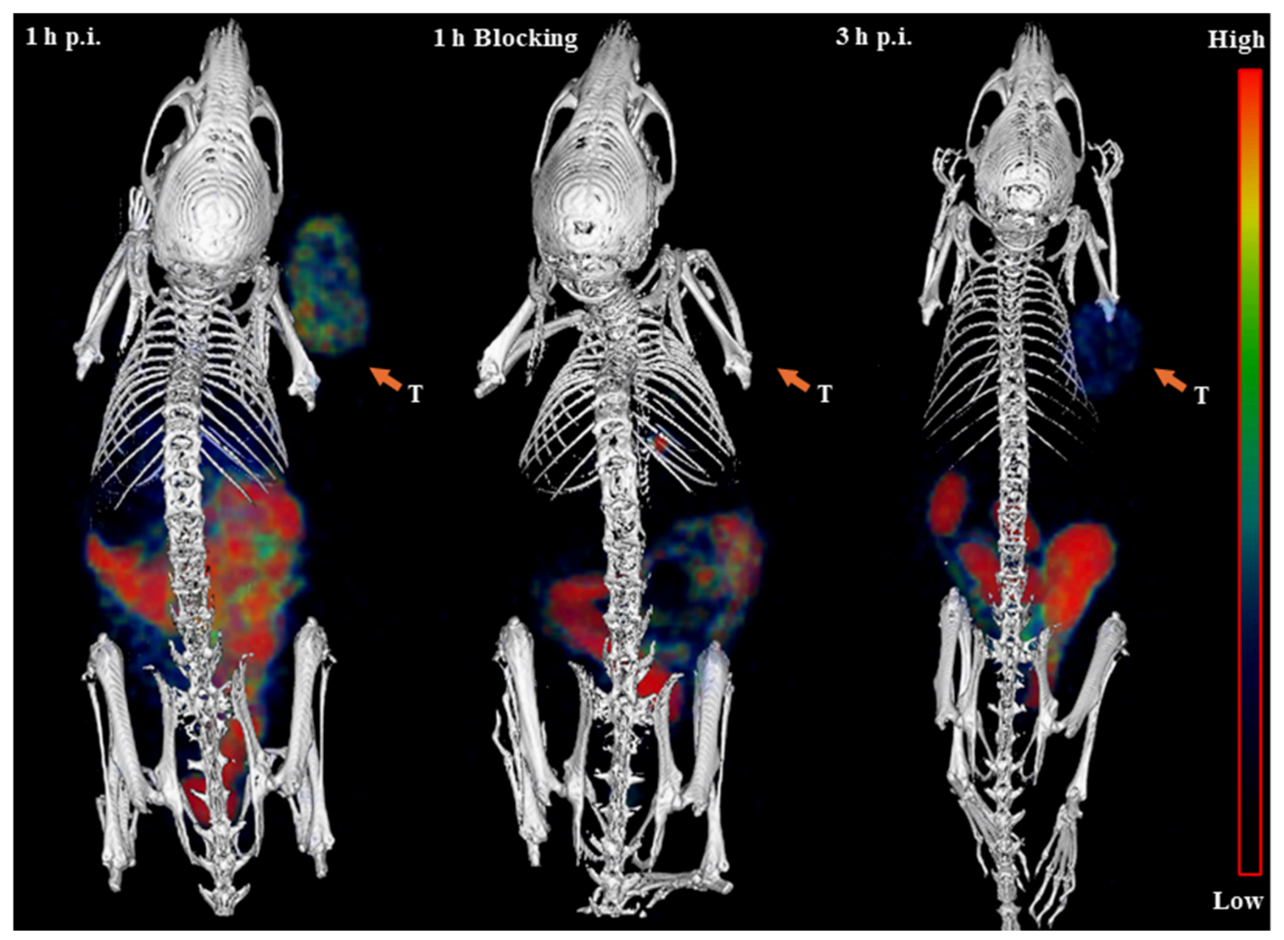
Representative SPECT/CT images of PC-3 xenograft-bearing mice (*n* = 3) after intravenous injection of ^99m^Tc2. Images were acquired at 1 h, 1 h blocking and 3 h p.i. The animals received approximately 30 MBq of radiotracer, and static SPECT acquisition was performed for 20 min. The PC-3 xenograft in the right shoulder/upper-flank region, indicated by arrows and the label T, is visualized in the non-blocked images, whereas tumor uptake is markedly suppressed under blocking conditions. Prominent abdominal activity reflects hepatobiliary/intestinal excretion with renal/urinary contribution. Images are presented as maximum intensity projections. The head is oriented superiorly.

Static SPECT/CT imaging was performed in PC-3 xenograft-bearing mice at 1 h and 3 h p.i. using Molecubes γ-CUBE/X-CUBE systems (Figure 9). At 1 h p.i., the PC-3 xenograft in the right shoulder/upper-flank region was clearly visualized as a discrete focus of uptake above the surrounding soft-tissue background. The tumor signal was lower than the dominant abdominal excretory activity but remained clearly distinguishable from adjacent muscle. Prominent abdominal activity was observed, consistent with hepatobiliary/intestinal excretion and renal/urinary excretion. Low activity was visually detected in the thoracic region, head, and skeletal structures, consistent with the low ex vivo uptake in blood, lung, brain, muscle, and bone.

At 3 h p.i., background activity was further reduced, allowing assessment of tumor-to-background contrast at a later time point. In the blocking imaging study, tumor visualization was markedly suppressed, confirming that the visualized tumor signal was GRPR-mediated. A weak upper abdominal or peripancreatic signal was visible in the non-blocked 1 h image; however, precise assignment to the pancreas was limited on maximum-intensity projection images due to overlap with adjacent abdominal structures. Under blocking conditions, this receptor-associated abdominal component appeared reduced, while intestinal and urinary activity remained visible.

SPECT and CT datasets were co-registered and fused using Imalytics Preclinical software v3.1.6. Images are presented as maximum intensity projections.

## 3. Discussion

The present study describes the development of a GRPR-targeted ^99m^Tc/^188^Re theranostic pair based on NOTA-P2-RM26. The diagnostic analog, ^99m^Tc2, was successfully prepared using the *fac*-[^99m^Tc(CO)_3_]^+^ core after systematically optimizing the radiolabeling conditions. The best labeling performance was obtained with 100 μg NOTA-P2-RM26 in 0.1 M sodium acetate buffer at pH 5, incubated at 75 °C for 30 min. Under these conditions, radiochemical conversion reached approximately 85%, and after HPLC purification and SPE-based reformulation, the final product had radiochemical purity above 97%. Although HPLC purification currently limits direct kit-type preparation, it ensures the removal of unreacted precursor and potential side products before biological evaluation.

The use of the *fac*-[M(CO)_3_]^+^ core is attractive for the development of matched technetium/rhenium radiopharmaceuticals because technetium and rhenium share closely related organometallic coordination chemistry [29,30,36]. The *fac*-[^188^Re(CO)_3_]^+^ core has also been explored for biomolecule labeling using different donor systems, including histidine-rich peptide sequences and dipicolylamine-based chelators [37,38]. In this work, the non-radioactive rhenium ^nat^Re2 was synthesized and used as a reference compound for HPLC comparison with the radioactive complexes. Complex ^188^Re2 was prepared under the optimized labeling conditions developed for ^99m^Tc2 and exhibited hydrophilic behavior and a stability profile comparable to that of ^99m^Tc2, supporting the chemical feasibility of a matched diagnostic/therapeutic pair. The stabilities of ^99m^Tc2 and ^188^Re2 under challenge conditions are consistent with the known importance of ligand denticity and coordination environment for the kinetic inertness of tricarbonyl complexes [39]. However, because the current ^188^Re evaluation was limited to radiolabeling, stability, and lipophilicity, further in vivo biodistribution, dosimetry, and therapy studies are required before therapeutic performance can be assessed [25]. The importance of experimentally validating matched ^99m^Tc/^188^Re pairs has recently been highlighted in the case of maSSS/maSES-PEG_2_-RM26. In that study, the ^188^Re-labeled analogs showed biodistribution patterns closely resembling those of their ^99^ᵐTc counterparts, supporting the use of ^99m^Tc imaging agents for dosimetry estimation of ^188^Re therapeutic analogs. However, the authors also observed lower tumor uptake with the ^99m^Tc analogs, which may lead to an underestimation of the absorbed dose in the tumor [20]. Although rhenium is similar to technetium, they are not identical [40].

The in vitro data confirmed a GRPR-mediated mechanism of cell association. In PC-3 cells, ^99m^Tc2 showed high uptake, reaching 20.0 ± 1.0% of the added activity after 1 h of incubation. A GRPR-negative or GRPR-low cell line was not included; such a model would provide an additional orthogonal control. Nevertheless, co-incubation with excess non-radioactive NOTA-P2-RM26 reduced uptake to 1.4 ± 0.3%, corresponding to approximately 93% inhibition. This strong reduction supports specific binding to GRPR-expressing PC-3 cells. The internalization profile also showed that most cell-associated activity remained membrane-bound, with a comparatively low internalized fraction. This behavior is consistent with the pharmacological profile of RM26-based GRPR antagonists, which are expected to show strong receptor binding with limited internalization [7,8,10,11,12]. A similar membrane-dominant profile was reported for ^188^Re-labeled maSSS/maSES-PEG_2_-RM26 analogs, in which most of the cell-associated activity remained bound to the membrane, with only a small fraction slowly internalized over time [20]. Therefore, the low internalized fraction should not be interpreted as poor target engagement, but rather as a characteristic feature of the antagonist scaffold.

**Figure 10:**
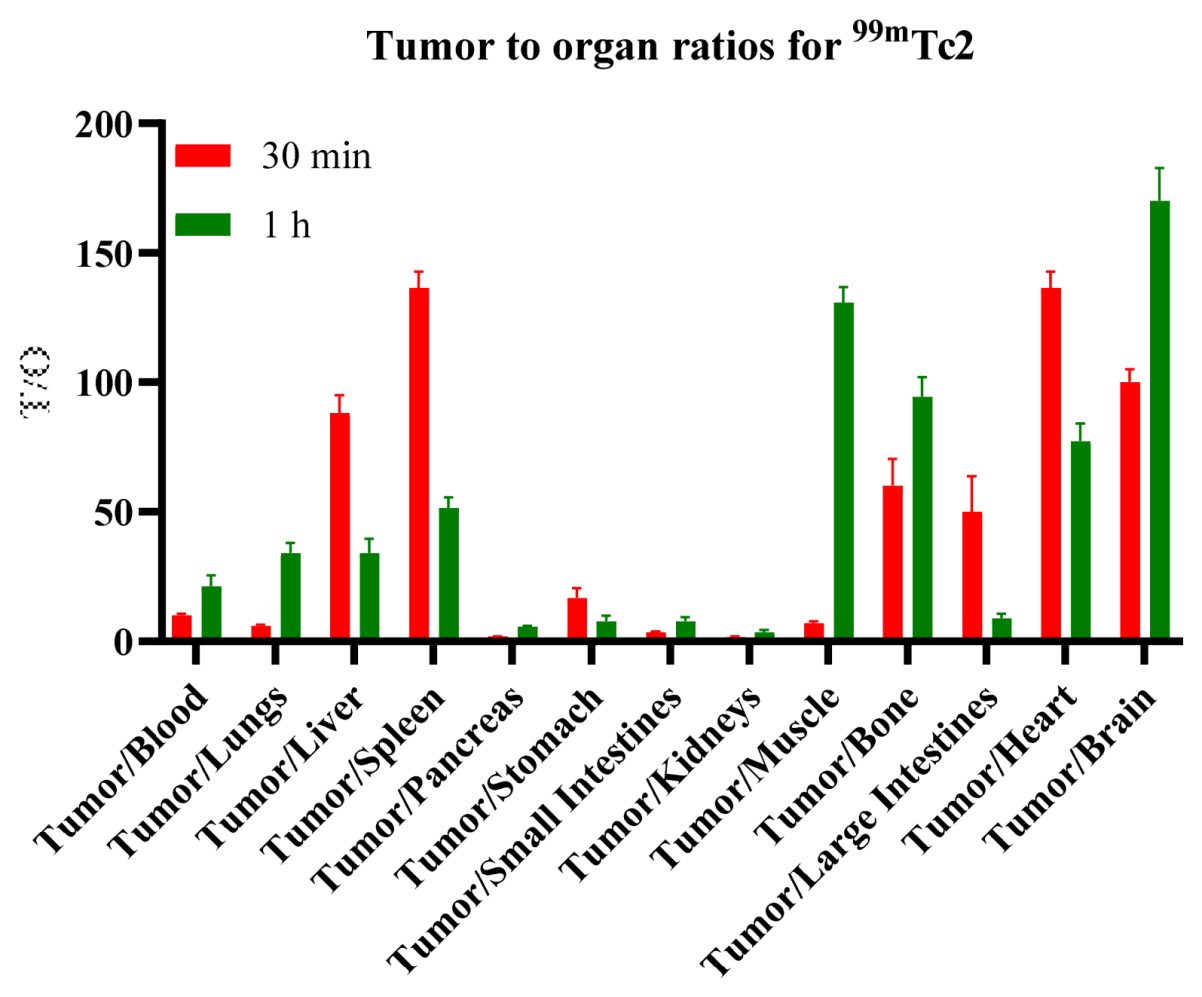
Tumor-to-organ ratios for ^99m^Tc2 were calculated from biodistribution data.

The biodistribution data showed high, sustained tumor uptake in PC-3 xenograft-bearing mice. Tumor accumulation reached 15.0 ± 1.0 %IA/g at 30 min and increased slightly to 17.0 ± 2.2 %IA/g at 1 h p.i. At the same time, blood and several non-target tissues cleared rapidly. Blood activity decreased from 1.5 ± 0.3 %IA/g at 30 min to 0.8 ± 0.13 %IA/g at 1 h p.i., and uptake in liver, spleen, muscle, bone, heart, brain, fat, and skin remained low at 1 h. This resulted in favorable tumor-to-background ratios, particularly tumor-to-blood and tumor-to-muscle ratios of approximately 21.3 and 130.8, respectively, at 1 h p.i. These ratios are consistent with the clear tumor delineation observed by SPECT/CT imaging (Figure 10).

The excretion profile of ^99m^Tc2 indicates predominant hepatobiliary/intestinal elimination, with a renal contribution. Although increased hydrophilicity is often sought to reduce nonspecific hepatobiliary retention in ^99m^Tc(CO)_3_ radiopharmaceuticals, the present biodistribution data indicate that the overall chelator–linker–peptide architecture still strongly influences clearance [41]. The predominance of hepatobiliary/intestinal excretion represents the main pharmacokinetic limitation of ^99m^Tc2. Similar considerations were raised for ^188^Re-labeled maSSS/maSES-PEG2-RM26, where high gastrointestinal activity was identified as a factor requiring further structural optimization [20]. Future linker or chelator modifications may be needed to reduce intestinal retention while preserving the high tumor uptake and favorable tumor-to-background ratios observed in the present study. Kidney uptake was relatively high at 30 min, reaching 8.5 ± 0.4 %IA/g, but decreased to 4.7 ± 0.22 %IA/g at 1 h p.i. Urine/bladder content increased from 3.5%IA at 30 min to 7.5%IA at 1 h, indicating renal clearance of part of the injected activity. However, gastrointestinal content accounted for the largest recovered fraction, increasing from 67% IA at 30 min to 82% IA at 1 h. In addition, small-intestine uptake was evident at 30 min, and large-intestine uptake increased by 1 h, supporting hepatobiliary/intestinal elimination as the main clearance route. This clearance profile should be carefully considered in future development, especially for imaging of lesions near the abdomen or pelvis and for dosimetry of the ^188^Re2. Such behavior is consistent with previous observations that molecular charge and chelator/linker architecture can markedly influence the biodistribution of ^99m^Tc(CO)_3_-labeled bombesin analogs [42,43].

A particularly relevant finding was the relationship between tumor and pancreatic uptake. The pancreas is a physiologically GRPR-expressing organ in mice and commonly shows prominent uptake of GRPR-targeted radioligands. In the present study, pancreatic uptake was elevated at 30 min, reaching 8.0 ± 0.4 %IA/g, but decreased to 3.0 ± 0.35 %IA/g at 1 h p.i. In contrast, tumor uptake remained high over the same interval. As a result, the tumor-to-pancreas ratio improved from approximately 1.9 at 30 min to approximately 5.7 at 1 h p.i. This is an important feature for GRPR-targeted imaging because high pancreatic uptake can limit tumor-to-pancreas contrast in preclinical mouse models.

Compared with previously reported tricarbonyl GRPR-targeted radioconjugates, ^99m^Tc2 showed a favorable tumor-to-pancreas profile. Makris et al. reported ^99m^Tc-labeled NOTA/NODAGA GRPR antagonist bioconjugates with pancreatic uptake values of approximately 15–24 %IA/g at 1 h p.i. and tumor uptake values of 4.3 ± 0.7 and 8.3 ± 0.9 %IA/g in PC-3 xenograft-bearing mice [32]. In comparison, ^99m^Tc2 showed tumor uptake of 17.0 ± 2.2 %IA/g and pancreatic uptake of 3.0 ± 0.35 %IA/g at 1 h p.i. This suggests improved tumor-to-pancreas contrast for the present tracer. Direct comparisons should still be made with caution because compound structures, injected peptide masses, formulations, animal model conditions, and experimental protocols differ between studies. Previous ^99m^Tc(CO)_3_-bombesin studies have also shown that linker, chelator, charge, and hydrophilicity can strongly influence biodistribution and clearance [44].

The in vivo blocking study provided strong evidence for receptor-mediated uptake. Administration of excess NOTA-P2-RM26 reduced tumor uptake from 17.0 ± 2.2 %IA/g to 0.84 ± 0.12 %IA/g, corresponding to approximately 95% inhibition. Pancreatic uptake was also reduced from 3.0 ± 0.35 %IA/g to 0.20 ± 0.08 %IA/g, corresponding to approximately 93% inhibition. Uptake in other GRPR-expressing tissues, including the stomach and small intestines, was also reduced under blocking conditions. In contrast, activity in non-target tissues remained low, and kidney uptake was only partially reduced, indicating that renal activity is primarily attributable to tracer clearance rather than to GRPR-specific binding. These findings confirm that uptake in both the tumor and the pancreas is predominantly receptor-mediated.

The imaging results were consistent with the ex vivo biodistribution data. Dynamic γ-eye imaging showed early tracer distribution followed by clearance, with visible tumor-associated activity and prominent abdominal activity. Static SPECT/CT imaging at 1 h p.i. confirmed visualization of the PC-3 xenograft as a discrete focus above the surrounding soft-tissue background. The tumor signal was lower than that of the dominant abdominal excretory activity, but it remained clearly distinguishable from the adjacent muscle. In the blocking SPECT/CT study, tumor visualization was markedly suppressed, supporting the receptor-mediated origin of the tumor signal. The non-blocked images also showed upper abdominal activity that may include a receptor-mediated pancreatic component, although definitive assignment to the pancreas is limited on maximum-intensity-projection images due to overlap with adjacent abdominal organs. Under blocking conditions, this receptor-associated component was reduced, whereas intestinal and urinary activity remained visible, consistent with the clearance pattern observed in the biodistribution study.

## 4. Materials and Methods

### 4.1. General Materials and Instrumentation

NOTA-P2-RM26 was obtained from MedChemExpress (Monmouth Junction, NJ, USA) and used without further purification unless otherwise stated. Sodium pertechnetate, Na[^99m^Tc]TcO_4_, was eluted in 0.9% saline from an Ultra-TechneKow™ V4 ^99^Mo/^99m^Tc generator (Curium Pharma, Petten, The Netherlands). Carrier-free Na[^188^Re]ReO_4_ was obtained from a ^188^W/^188^Re generator (OncoBeta GmbH, Garching, Germany). All other reagents and solvents were of analytical or HPLC grade and were purchased from commercial suppliers.

Analytical UPLC-QToF-MS was performed using a Waters ACQUITY UPLC I-Class system coupled to a Waters Xevo QTof mass spectrometer equipped with an electrospray ionization source operated in positive-ion mode. Chromatographic separation was performed on a Waters ACQUITY UPLC Peptide CSH C18 column, 130 Å, 1.7 µm, 2.1 × 150 mm. Mobile phase A was water/acetonitrile, 90:10 *v*/*v* (0.1% formic acid, FA), and mobile phase B was acetonitrile (0.1% FA). The flow rate was 0.400 mL/min. The gradient was 0–10.0 min, 0–100% B; 10.0–12.5 min, 100% B; 12.5–13.0 min, 100–0% B; and 13.0–15.0 min, 0% B. The injection volume was 10 µL, the column temperature was 45 °C, and the autosampler temperature was 20 °C. The mass spectrometer was operated in ESI positive-ion mode with the following source parameters: capillary voltage, 1.0 kV; sampling cone, 30 V; extraction cone, 6 V; source temperature, 120 °C; desolvation temperature, 550 °C; cone gas flow, 1 L/h; and desolvation gas flow, 700 L/h. Data were acquired over an *m*/*z* range of 100–2000 using continuum acquisition. Data were acquired and processed using MassLynx v4.1.

Analytical and semi-preparative HPLC analyses were performed using a Waters Alliance e2695 HPLC system equipped with a Waters 2487 dual-wavelength absorbance detector (Waters Corporation, Milford, MA, USA) and a Gabi Star NaI radioactivity detector (Elysia-Raytest, Straubenhardt, Germany). Analytical HPLC was performed on an EC Zorbax Eclipse Plus C18 column (4.6 × 100 mm, 3 μm; Agilent Technologies, Santa Clara, CA, USA). The analytical HPLC mobile phase consisted of water containing 0.1% trifluoroacetic acid (TFA) and acetonitrile containing 0.1% TFA. The gradient started at 90:10 (*v*/*v*) water/acetonitrile and was held for 1 min, then increased linearly to 40:60 (*v*/*v*) water/acetonitrile at 9 min. A rapid gradient to 10:90 was then applied at 9.1 min and held until 12 min to elute strongly retained species. The system was returned to its initial conditions at 12.1 min to re-equilibrate. The flow rate was maintained at 1.2 mL/min. UV detection was performed at 254 nm, and radioactivity was monitored using the in-line radioactivity detector.

Radio-TLC analysis was performed using iTLC-SG strips developed with saline or saline/methanol (1:1, *v*/*v*). TLC measurements were performed using the γ-eye™ scintigraphic desktop scanner (Bioemtech, Athens, Greece). The system provides a field of view (FOV) of 50 × 100 mm, enabling Radio-TLC measurements. TLC strips were placed on the scanner bed platform, and acquisitions were performed for 2 min. We reconstructed chromatographic profiles using visual|eyes (Bioemtech). Radioactivity measurements from biological samples were performed using a Wallac Wizard 1470 gamma counter (PerkinElmer, Waltham, MA, USA). When required for non-radioactive material, flash chromatography was performed using a Reveleris X2 Flash Chromatography System (Grace Discovery Sciences, Columbia, MD, USA).

### 4.2. Preparation of the Rhenium and Technetium Tricarbonyl Precursors

The non-radioactive rhenium tricarbonyl precursor, *fac*-[Et_4_N]_2_[ReBr_3_(CO)_3_], ^nat^Re1, was prepared from dirhenium decacarbonyl according to previously reported procedures [45]. The *fac*-[^99m^Tc][Tc(CO)_3_(H_2_O)_3_]^+^, ^99m^Tc1 precursor, was prepared according to previously reported procedures with minor modifications [46]. Briefly, Na[^99m^Tc]TcO_4_ (1.0 mL, 370–720 MBq) in 0.9% NaCl was added to a sealed CO-saturated vial containing NaBH_4_ (5.5 mg), Na_2_CO_3_ (4 mg), and sodium potassium tartrate (10 mg). The reaction mixture was heated at 85 °C for 30 min. After cooling to room temperature, the pH was adjusted to approximately 7.0 with 1 M HCl. The formation of ^99m^Tc1 was confirmed by radio-HPLC, affording the precursor in >93% radiochemical yield and exhibiting a retention time of approximately 3.2 min. The *fac*-[^188^Re][Re(CO)_3_(H_2_O)_3_]^+^, ^188^Re1, precursor, was prepared from Na[^188^Re]ReO_4_ according to established literature procedures [30,35]. Briefly, Na[^188^Re]ReO_4_ (74–100 MBq) in saline (1 mL), acidified with 85% H_3_PO_4_ (7 μL), was added to a sealed 10 mL serum vial containing BH_3_NH_3_ (5 mg), previously purged with CO. The vial was heated at 60 °C for 20 min, and the evolving gas pressure was balanced using a syringe. The vial was then cooled in an ice bath, and the pH was adjusted to approximately 5.5. Radio-HPLC confirmed the formation of ^188^Re1, affording the precursor in >90% radiochemical yield, with a retention time of approximately 3.2 min. The precursor was used directly for radiolabeling without further purification.

### 4.3. Synthesis of Fac-[^nat^Re(CO)_3_(NOTA-P2-RM26)], ^nat^Re2

The non-radioactive rhenium, ^nat^Re2, was synthesized using the corresponding rhenium tricarbonyl precursor ^nat^Re1. NOTA-P2-RM26 (5 μmol) was dissolved in 0.1 M sodium acetate buffer (2 mL, pH 5) and reacted with ^nat^Re1 (6 μmol). The reaction mixture was heated at 80 °C for 4 h. The crude mixture was analyzed by analytical HPLC, and the product peak corresponding to ^nat^Re2 was observed. The desired product was purified by semi-preparative reversed-phase HPLC using a Zorbax Eclipse Plus C18 column (9.4 × 250 mm, 5 μm). The mobile phase consisted of water containing 0.1% TFA (A) and acetonitrile containing 0.1% TFA (B). The gradient started at 95:5 (*v*/*v*) A/B and held for 2 min, then increased linearly to 60:40 over 20 min to separate the metalated peptide from unreacted precursor and side products. A rapid increase to 10:90 was then applied at 22 min and maintained until 25 min to elute strongly retained species, after which the system was returned to 95:5 at 26 min for re-equilibration. The flow rate was 4 mL/min. UV detection was performed at 254 nm. The product fraction was collected based on the UV chromatogram and subsequently analyzed by analytical HPLC and mass spectrometry. UPLC-QToF-MS, ESI^+^: *m*/*z* 906.7042, 907.2195, 907.6968, 908.2123, 908.7090, 909.2057, and 909.7217 (observed doubly charged isotope envelope, consistent with [M+2H]^2+^; isotope spacing approximately 0.5 *m*/*z*; Figure 1). The purified ^nat^Re2 was used as a reference standard for comparative HPLC analysis of ^99m^Tc2 and ^188^Re2.

### 4.4. Radiolabeling Optimization of Fac-[^99m^Tc][Tc(CO)_3_(NOTA-P2-RM26)], ^99m^Tc2

Radiolabeling of NOTA-P2-RM26 with the *fac*-[^99m^Tc(CO)_3_]^+^ core was optimized by varying peptide amount, reaction temperature, incubation time, pH, and buffer composition. NOTA-P2-RM26 was tested at 1, 10, and 100 μg. Reactions were performed at room temperature, 75 °C, or 100 °C. Incubation times of 15, 30, and 60 min were evaluated. The effect of pH and reaction medium was assessed using 0.1 M sodium acetate buffer at pH 5, 0.1 M phosphate buffer at pH 7 or 9, and non-buffered conditions. All reactions were performed under nitrogen. The optimized radiolabeling procedure consisted of reacting NOTA-P2-RM26 (100 μg, 100 μL) with freshly prepared ^99m^Tc1 (400 μL) in 0.1 M sodium acetate buffer at pH 5. The reaction mixture was incubated at 75 °C for 30 min. Under these conditions, ^99m^Tc2 was obtained with a radiochemical conversion of approximately 85%, as determined by radio-HPLC analysis of the crude reaction mixture.

### 4.5. Purification, Spe Reformulation, and Quality Control of ^99m^Tc2

After radiolabeling, the crude reaction mixture was purified by radio-HPLC. The radioactive fraction corresponding to ^99m^Tc2 was collected and diluted to a final volume of 5 mL with water prior to solid-phase extraction. An HLB cartridge was conditioned with 4 mL of ethanol and 8 mL of water before loading the collected HPLC fraction. After loading, the cartridge was washed with 2 mL of water to remove residual buffer and HPLC solvents. The retained radioconjugate was eluted with 1 mL of ethanol and diluted with saline to obtain the final injectable formulation containing 10% ethanol (*v*/*v*). The final formulation was analyzed by radio-HPLC and radio-TLC. Radiochemical purity was required to be >95% before use in biological experiments. The purified radioconjugate typically had radiochemical purity > 97%.

### 4.6. Radiolabeling and Evaluation of Fac-[^188^Re][Re(CO)_3_(NOTA-P2-RM26)], ^188^Re2

Complex ^188^Re2 was prepared using the optimized radiolabeling conditions developed for ^99m^Tc2. Briefly, NOTA-P2-RM26 (100 μg) was reacted with freshly prepared ^188^Re1 in 0.1 M sodium acetate buffer at pH 5. The reaction mixture was incubated at 75 °C for 30 min. The resulting ^188^Re2 product was analyzed by radio-HPLC and compared with the ^nat^Re2 reference compound. Radiochemical conversion, radiochemical purity, in vitro stability, and logD_7.4_ were determined using procedures analogous to those applied for ^99m^Tc2.

### 4.7. In Vitro Stability Studies

The in vitro stability of ^99m^Tc2 and ^188^Re2 was evaluated in the final formulation, in human serum, and in the presence of cysteine and histidine [47]. To assess formulation stability, aliquots of the purified radioconjugate, formulated in saline containing 10% ethanol (*v*/*v*), were incubated at 37 °C and analyzed by radio-HPLC at predetermined time points up to 6 h. For serum stability, aliquots of the radioconjugate were incubated in human serum at 37 °C. At selected time points, samples were treated with acetonitrile or ethanol to precipitate serum proteins. The samples were centrifuged, and the supernatant was analyzed by radio-HPLC. Challenge studies were performed by mixing purified radioconjugate (0.5 mL, approximately 3.7 MBq) with an equal volume of cysteine or histidine solution (0.5 mL, 2 × 10^−2^ M in water). Samples were incubated at 37 °C and analyzed by radio-HPLC at 1, 3, and 6 h. Stability results are presented as mean ± SD from independent experiments (*n* = 3 for ^99^ᵐTc2 and ^188^Re2).

### 4.8. Determination of Distribution Coefficient LogD_7.4_

The lipophilicity of the radiolabeled complexes was determined by the shake-flask method [48]. Briefly, the radioconjugate (approximately 74 kBq in 50 μL) was added to a biphasic mixture of n-octanol (1.5 mL) and PBS (1.5 mL, 0.1 M, pH 7.4). The mixture was vortexed for 1 min at room temperature and centrifuged at 5000 rpm for 5 min to ensure phase separation. 100 μL aliquots were collected from each phase and measured with a gamma counter. The distribution coefficient was calculated as the ratio of radioactivity in the octanol phase to that in the aqueous phase, expressed as logD_7.4_. Measurements were performed in triplicate and repeated in three independent experiments. Data are reported as mean ± standard deviation.

### 4.9. Cell Culture

PC-3 human prostate cancer cells were cultured in F-12K (Kaighn’s modification of Ham’s F-12 medium) medium supplemented with L-glutamine, 10% fetal bovine serum, and 1% penicillin–streptomycin solution containing 10,000 U/mL penicillin and 10,000 μg/mL streptomycin. Cells were maintained in a humidified atmosphere at 37 °C and 5% CO_2_ using an HF90 incubator (Heal Force Bio-Meditech Holdings Limited, Shanghai, China). Cells were subcultured at approximately 70–80% confluency using 0.25% trypsin–EDTA solution.

### 4.10. Cellular Uptake and Internalization Studies

For cellular uptake and internalization studies, PC-3 cells were seeded in six-well plates at a density of 1 × 10^6^ cells per well and allowed to adhere overnight. On the day of the experiment, cells were washed once with 1 mL PBS at room temperature. Cells were incubated with complete medium containing approximately 100 kBq of ^99m^Tc2 at 37 °C for predetermined time points. At each time point, the incubation medium was removed, and cells were washed with 1 mL of ice-cold PBS to stop further uptake and internalization.

To determine the membrane-bound fraction, cells were incubated twice with 600 μL of acid-wash buffer (0.2 M glycine, 0.15 M NaCl, and 4 M urea) at pH 2 for 5 min on ice. The acid-wash fractions were collected and designated as the membrane-bound fraction. After acid washing, cells were washed once with PBS and lysed twice with 600 μL of 1 M NaOH. The lysates were collected and designated as the internalized fraction. Radioactivity in all collected fractions was measured using the gamma counter. Results were expressed as a percentage of added activity or percentage of maximum associated activity, as appropriate.

### 4.11. In Vitro Specificity Studies

For in vitro blocking studies, PC-3 cells were seeded as described above. After washing with PBS, cells were incubated for 1 h at 37 °C with complete medium containing approximately 100 kBq of ^99m^Tc2. For blocked samples, non-radioactive NOTA-P2-RM26 was added as a GRPR-blocking agent at a final concentration of 25 nM. After incubation, the medium was discarded, and the cells were detached using trypsin–EDTA. The cell suspension was collected into 5-mL tubes, and radioactivity was measured using a gamma counter. The degree of blocking was calculated by comparing cell-associated activity in blocked and non-blocked samples.

### 4.12. Animal Model

All animal experiments were conducted in accordance with national legislation and European Directive 2010/63/EU for the protection of animals used for scientific purposes. PC-3 xenografts were established in female Swiss Albino SCID mice, aged 7–8 weeks. After acclimatization, animals were injected subcutaneously into the right shoulder/upper-flank region with freshly harvested PC-3 cells suspended in PBS. Each animal received 5 × 10^6^ cells. Tumors were allowed to grow for approximately three weeks until palpable solid tumors were formed. At the time of the experiments, tumor volumes ranged from 200 to 400 mm^3^. Animals were then randomly assigned to experimental groups.

### 4.13. Biodistribution and In Vivo Blocking Studies

Biodistribution studies were performed at 30 min and 1 h post injection (p.i.). PC-3 xenograft-bearing mice received an intravenous injection via the tail vein of ^99m^Tc2 formulated in saline containing 10% ethanol (*v*/*v*). The injected volume was 100 μL, and the injected activity was approximately 100 kBq per animal. Animals weighed 21 ± 2 g on the day of the experiment. For in vivo blocking studies, a separate group of animals received non-radioactive NOTA-P2-RM26 (50 μg) 5 min prior to the radiotracer injection. Blocking animals were euthanized at 1 h p.i. At the selected time points, animals were euthanized. Blood, tumors, organs, and tissues of interest were collected, weighed, and measured for radioactivity using the gamma counter. The stomach and intestines were measured without content. Urine/bladder content and gastrointestinal/fecal content were collected when available and reported separately. Data were decay-corrected and expressed as a percentage of injected activity per gram of tissue (%IA/g). Urine/bladder and gastrointestinal/fecal content were expressed as a percentage of injected activity (%IA).

### 4.14. Imaging Studies

Dynamic planar imaging was performed using the γ-eye™ system (Bioemtech, Athens, Greece). PC-3 xenograft-bearing mice were injected intravenously with approximately 30 MBq of ^99m^Tc2 and imaged dynamically for up to 1 h p.i. The acquisitions were used to assess early tracer distribution, clearance, and tumor-associated activity. Blocking imaging was performed under the same conditions after administration of non-radioactive NOTA-P2-RM26 (50 μg) 5 min before radiotracer injection.

Static small-animal SPECT/CT imaging was performed at 1 h and 3 h p.i. using a γ-CUBE SPECT scanner and an X-CUBE CT scanner (Molecubes, Ghent, Belgium). Animals were anesthetized with isoflurane in oxygen, using 5% isoflurane for induction and 2.5% for maintenance at a flow rate of 1 L/min. Mice were positioned head-first, prone, on the imaging bed before scanning. Respiration and body temperature were monitored throughout the imaging session. Static SPECT acquisitions were performed for 20 min. Following SPECT acquisition, CT scans were acquired for anatomical co-registration using the X-CUBE CT scanner with the “General Purpose” protocol: 50 kVp, 480 exposures, 85 ms/projection, 100 μA tube current, and 60 s rotation time.

Dynamic datasets were reconstructed using Bioemtech software. Static SPECT and CT datasets were reconstructed using Cubeflow 2 (Molecubes). Post-analysis of the scans was performed using Imalytics Preclinical v5.1 software (Gremse-IT GmbH, Aachen, Germany) [49,50]. Fused SPECT/CT images and coronal maximum intensity projections were generated to visualize tracer distribution and anatomical localization. Where applicable, volumes of interest were manually delineated on fused SPECT/CT datasets for semi-quantitative assessment of tracer accumulation.

Blocking SPECT/CT images were acquired at 1 h p.i. using the same workflow for acquisition, reconstruction, fusion, and analysis.

### 4.15. Statistical Analysis

Data are presented as mean ± SD unless otherwise stated. Cellular assays were performed in three independent experiments. Biodistribution groups comprised four mice per condition, and imaging was performed in three mice. The specificity assay was analyzed using an unpaired two-tailed Student’s *t*-test. Biodistribution data were analyzed using two-way ANOVA followed by Tukey’s multiple-comparison test. Imaging findings were evaluated descriptively; the dynamic tumor time-activity curve is presented as mean ± SD for three mice. Statistical analyses were performed using GraphPad Prism 10.6.1 (GraphPad Software, Boston, MA, USA). A *p*-value < 0.05 was considered statistically significant.

## 5. Conclusions

This study reports the development of a GRPR-targeted ^99m^Tc/^188^Re theranostic pair based on NOTA-P2-RM26. The diagnostic complex ^99m^Tc2 was efficiently prepared using the *fac*-[^99m^Tc(CO)_3_]^+^ core, with optimized labeling conditions that afforded high radiochemical conversion and purity after purification and reformulation. The purified radioconjugate showed suitable in vitro stability and displayed a hydrophilic profile. In vitro studies in PC-3 cells confirmed GRPR-mediated binding, as uptake decreased in the presence of a blocking agent. The cell-association profile was dominated by the membrane-bound fraction, consistent with the expected behavior of an RM26-based GRPR antagonist. In PC-3 xenograft-bearing mice, ^99m^Tc2 demonstrated high tumor uptake, which was strongly reduced after blocking with excess NOTA-P2-RM26, confirming receptor-mediated tumor accumulation. Pancreatic uptake, consistent with physiological GRPR expression in mice, decreased rapidly and was minimized under blocking conditions, indicating specific uptake in both tumor and pancreas, with a favorable tumor-to-pancreas contrast. Dynamic γ-eye imaging and SPECT/CT confirmed tumor visualization and suppression of tumor signal under blocking conditions. The main limitation of ^99m^Tc2 was its predominant hepatobiliary/intestinal excretion, which should be considered in future optimization and in dosimetry studies for the ^188^Re2 complex, which showed comparable physicochemical behavior, supporting the feasibility of a chemically matched ^99m^Tc/^188^Re NOTA-P2-RM26 platform. Further studies, including biodistribution of ^188^Re2, dosimetry, and evaluation of therapeutic efficacy, are required to establish the full theranostic potential of this pair.

## Figures and Tables

**Table 1:** Optimization of radiolabeling conditions for ^99m^Tc2.

Trial	NOTA-P2-RM26 (μg)	Buffer/Medium	pH	Temperature (°C)	Time (min)	%RCC *
A1	1	0.1 M NaOAc	5	75	30	2
A2	10	0.1 M NaOAc	5	75	30	28
A3	100	0.1 M NaOAc	5	75	30	84
B1	100	0.1 M NaOAc	5	25	30	10
B2	100	0.1 M NaOAc	5	75	30	85
B3	100	0.1 M NaOAc	5	100	30	80
C1	100	0.1 M NaOAc	5	75	15	62
C2	100	0.1 M NaOAc	5	75	30	85
C3	100	0.1 M NaOAc	5	75	60	88
D1	100	0.1 M NaOAc	5	75	30	87
D2	100	0.1 M PBS	7	75	30	72
D3	100	0.1 M PBS	9	75	30	35
D4	100	No buffer	6	75	30	80

* RCC = radiochemical conversion, determined by radio-HPLC analysis of the crude reaction mixture before purification. The optimized condition was 100 μg NOTA-P2-RM26 in 0.1 M sodium acetate buffer at pH 5, incubated with freshly prepared ^99m^Tc1 at 75 °C for 30 min under nitrogen.

**Table 2:** In vitro stability of ^99m^Tc2 under formulation and challenge conditions.

Conditions	RCP (%) *		
	1 h	3 h	6 h
Saline containing 10% ethanol (*v*/*v*)	99	97	93
Human serum, 37 °C	92	87	85
Cysteine solution, 20 mM, 37 °C	97	95	92
Histidine solution, 20 mM, 37 °C	95	89	85

* RCP = radiochemical purity, determined by radio-HPLC. We performed stability studies after incubating purified ^99m^Tc2 in formulation, human serum, cysteine or histidine at 37 °C.

**Table 3:** Radiochemical and physicochemical characterization of ^99m^Tc2 and ^188^Re2.

Parameter	^99m^Tc2	^188^Re2
Independent radiolabeling preparations, *n*	10	10
RCC (%)	approximately 85	approximately 80
Post-purification RCP (%)	>97	>95 in all preparations
logD_7.4_	−1.5 ± 0.2	−1.6 ± 0.2
Formulation RCP (%) (SD)	99 (1)/97 (2)/93 (4)	100 (0)/98 (2)/92 (4)
Human serum RCP (%) (SD)	92 (2)/87 (5)/85 (3)	94 (2)/85 (5)/83 (6)
Cysteine RCP (%) (SD)	97 (1)/95 (1)/92 (3)	96 (1)/90 (2)/85 (4)
Histidine RCP (%) (SD)	95 (3)/89 (5)/85 (4)	92 (2)/86 (5)/83 (4)

RCP = radiochemical purity, determined by radio-HPLC. We performed stability studies after incubating purified ^99m^Tc2 in formulation, human serum, cysteine or histidine at 37 °C. Stability results are presented as mean ± SD from 3 independent experiments.

## Data Availability

The data presented in this study are available within the article. Additional data may be made available from the corresponding author upon reasonable request.

## Abbreviations

GRPR	Gastrin-releasing peptide receptor
SPECT	Single-photon emission computed tomography
CT	Computed tomography
PET	Positron emission tomography
RM26	GRPR antagonist peptide sequence D-Phe-Gln-Trp-Ala-Val-Gly-His-Sta-Leu-NH_2_
NOTA	1,4,7-triazacyclononane-1,4,7-triacetic acid
P2	Diethylene glycol/PEG_2_ spacer
RCC	Radiochemical conversion
RCP	Radiochemical purity
RCY	Radiochemical yield
HPLC	High-performance liquid chromatography
TLC	Thin-layer chromatography
SPE	Solid-phase extraction
p.i.	Post injection
%IA/g	Percentage of injected activity per gram of tissue
%IA	Percentage of injected activity
MB	Membrane-bound fraction
Int	Internalized fraction
logD_7.4_	Distribution coefficient at pH 7.4
VOI	Volume of interest
NaOAc	Sodium acetate
PBS	Phosphate-buffered saline
TFA	Trifluoroacetic acid
FBS	Fetal bovine serum
